# Direct interaction with 14–3-3γ promotes surface expression of Best1 channel in astrocyte

**DOI:** 10.1186/s13041-017-0331-x

**Published:** 2017-11-09

**Authors:** Soo-Jin Oh, Junsung Woo, Young-Sun Lee, Minhee Cho, Eunju Kim, Nam-Chul Cho, Jae-Yong Park, Ae Nim Pae, C. Justin Lee, Eun Mi Hwang

**Affiliations:** 1 Center for Neuroscience, Korea Institute of Science and Technology, Seoul, Korea; 2 Convergence Research Center for Diagnosis, Treatment and Care System of Dementia, Korea Institute of Science and Technology, Seoul, Korea; 3 Center for Glia-Neuron interaction, Korea Institute of Science and Technology, Seoul, Korea; 4 Center for Functional Connectomics, Korea Institute of Science and Technology, Seoul, Korea; 5 Neuroscience Program, University of Science and Technology (UST), Daejeon, Korea; 6 School of Biosystem and Biomedical Science, College of Health Science, Korea University, Seoul, Korea

**Keywords:** Astrocyte, Bestrophin-1, 14–3-3γ, Surface expression, Glutamate

## Abstract

**Background:**

Bestrophin-1 (Best1) is a calcium-activated anion channel (CAAC) that is expressed broadly in mammalian tissues including the brain. We have previously reported that Best1 is expressed in hippocampal astrocytes at the distal peri-synaptic regions, called microdomains, right next to synaptic junctions, and that it disappears from the microdomains in Alzheimer’s disease mouse model. Although Best1 appears to be dynamically regulated, the mechanism of its regulation and modulation is poorly understood. It has been reported that a regulatory protein, 14-3-3 affects the surface expression of numerous membrane proteins in mammalian cells.

**Methods:**

The protein-protein interaction between Best1 and 14-3-3γ was confirmed by yeast-two hybrid assay and BiFC method. The effect of 14-3-3γ on Best1-mediated current was measured by whole-cell patch clamp technique.

**Results:**

We identified 14-3-3γ as novel binding partner of Best1 in astrocytes: among 7 isoforms of 14-3-3 protein, only 14-3-3γ was found to bind specifically. We determined a binding domain on the C-terminus of Best1 which is critical for an interaction with 14-3-3γ. We also revealed that interaction between Best1 and 14-3-3γ was mediated by phosphorylation of S358 in the C-terminus of Best1. We confirmed that surface expression of Best1 and Best1-mediated whole-cell current were significantly decreased after a gene-silencingof 14-3-3γ without a significant change in total Best1 expression in cultured astrocytes. Furthermore, we discovered that 14-3-3γ-shRNA reduced Best1-mediated glutamate release from hippocampal astrocyte by recording a PAR1 receptor-induced NMDA receptor-mediated current from CA1 pyramidal neurons in hippocampal slices injected with adenovirus carrying 14-3-3γ-shRNA. Finally, through a structural modeling, we found critical amino acid residues containing S358 of Best1 exhibiting binding affinities to 14-3-3γ.

**Conclusions:**

14-3-3γ promotes surface expression of Best1 channel in astrocytes through direct interaction.

## Introduction

Astrocytes provide structural and trophic support to neurons as well as an active interaction with neurons. It has been reported that astrocytes can be activated by a variety of physiological and pathological stimuli which can evoke increases in intracellular Ca^2+^ in astrocytes [1, 2]. Astrocytes, in turn, elicit the release of active substances called gliotransmitters to regulate neuronal activities [3–6]. Recently, several studies have shown that hippocampal astrocytes express machinery responsible for Ca^2+^-dependent and channel-mediated glutamate release, which is encoded by Best1 channel [7, 8]. *Bestrophin* is the gene responsible for a dominantly inherited, juvenile-onset form of macular degeneration called Best’s vitelliform macular dystrophy. It has been shown to encode a functional Ca^2+^-activated anion channel (CAAC) directly activated by submicromolar intracellular Ca^2+^ concentration in nonneuronal tissue and peripheral neurons [9]. Our previous studies indicate that Best1 has a permeability to gamma aminobutyric acid (GABA), which contributes majorly to the tonic form of neuronal inhibition [10]. We also demonstrated that Best1 releases glutamate, which targets and activates synaptic NMDA receptors in hippocampal CA1 pyramidal neurons [11] to modulate hippocampal synaptic plasticity [12].

This glutamate- and GABA-permeable Best1 is selectively expressed at the astrocytic microdomains adjacent to glutamatergic synapse by electron microscopy [13]. In our recent study, we observed that astrocytes around amyloid plaques in Alzheimer’s disease (AD) model mouse become reactive and produce GABA [14]. Meanwhile, Best1 channel is redistributed away from microdomains to the soma and processes of reactive astrocytes, possibly switching its target from synaptic NMDA receptors to extrasynaptic GABA receptors [14]. Therefore, regulation of surface expression and channel distribution of the glutamate- and GABA-permeable Best1 probably have critical roles in astrocyte-neuron interaction and regulation of synaptic functions. Despite accumulating lines of evidence that Best1 has significant roles in synaptic functions both in physiological condition and in pathological condition of the brain [15] and its surface expression is dynamically regulated, relatively little is known about its mechanisms of regulation and modulation, especially of the regulation of surface expression.

To further explore the regulatory mechanism of the surface expression of Best1, we set forth to identify novel binding partners of Best1. The 14–3-3 proteins are a family of conserved regulatory molecules expressed in all eukaryotic organisms [16]. They have ability to bind a multitude of functionally wide array of cellular proteins. Through interaction with its effector proteins, 14–3-3 proteins participate in vital regulatory processes such as neuronal development, apoptotic cell death, cell cycle control, viral and bacterial pathogenesis and cellular activity including the subcellular localization of target proteins [17, 18]. They are abundantly expressed in the brain and have been detected in the cerebrospinal fluid (CSF) of patients with the various neurological disorders [19]. Several studies demonstrated that 14–3-3 proteins, by binding to phosphorylated motifs, promote the plasma membrane expression of integral membrane proteins such as the nicotinic acetylcholine and GABA receptors [20, 21], potassium channels [22, 23], the epithelial Na^+^ channel, ENaC [24, 25] and TRPM4b channel [26]. These interactions between 14 and 3-3 proteins and various ion channels and possible role of the interaction in the pathophysiology of the brain suggest possible involvement of 14–3-3 protein in Best1 channel function. Therefore, we have employed biochemical and electrophysiological assays to investigate the potential protein-protein interaction between 14 and 3-3γ and Best1 channel. We demonstrate that 14–3-3γ promotes the surface expression of Best1 by interaction to C-terminus of Best1 in astrocytes.

## Methods

### Animals

Ca^2+^/calmodulin-dependent kinase IIα promoter-driven Cre (CaMKIIα-Cre) transgenic mice purchased from Jackson Laboratory were used for cell type specific gene silencing system. For electrophysiological experiments, 7 to 8-week-old male mice were used. Animal care and handling were performed according to the directives of the Animal Care and Use Committee and institutional guidelines of KIST (Seoul, Korea).

### Cell culture

HEK293T cells were cultured in Dulbecco’s modified Eagle’s medium (DMEM; Gibco) and COS7 cells were cultured in RPMI medium 1640 (Gibco) supplemented with 10% heat-inactivated fetal bovine serum and 1000 units ml^−1^ penicillin–streptomycin. Cultures were maintained at 37 °C in a humidified 5% CO_2_-containing atmosphere. For primary culture of cortical astrocytes, P0-P3 C57BL/6 J mice were used. The cerebral cortex was dissected free of adherent meninges, minced and dissociated into single cell suspension by trituration through a Pasteur pipette. Dissociated cells were plated onto either 12 mm glass coverslips or six-well plates coated with 0.1 mg ml^−1^ poly d-lysine. Cells were grown in DMEM supplemented with 25 mM glucose, 10% heat-inactivated horse serum, 10% heat-inactivated fetal bovine serum, 2 mM glutamine and 1000 units ml^−1^ penicillin–streptomycin. Cultures were maintained at 37 °C in a humidified 5% CO_2_-containing atmosphere. Astrocyte cultures prepared in this way were previously determined by glial fibrillary acidic protein (GFAP) staining to be greater than 95% astrocytes [27].

### Electrophysiological recording from cultured astrocytes

In whole-cell patch clamp, patch pipettes which have 3 ~ 6 MΩ of resistance are filled with the standard intracellular solution. Current voltage curves were established by applying 100-, 200-, or 1000 ms duration voltage ramps from −100 to +100 mV. Data were acquired by an Axopatch 200A amplifier controlled by Clampex 10.2 via Digidata 1322A data acquisition system (Axon Instruments). Experiments were conducted at room temperature (20 ~ 24 °C). To activate CAAC or Best1 directly, high Ca^2+^-containing intracellular patch pipette solution was applied to cultured astrocytes, which is comprised of 146 mM CsCl, 5 mM (Ca^2+^)-EGTA-NMDG, 2 mM MgCl_2_, 8 mM HEPES, and 10 mM Sucrose at pH 7.3, adjusted with NMDG. For control experiments, Ca^2+^-free intracellular solution comprised of 146 mM CsCl, 5 mM EGTA-NMDG, 2 mM MgCl_2_, 8 mM HEPES, and 10 mM Sucrose at pH 7.3, adjusted with NMDG was used. The concentration of free [Ca^2+^]_i_ in the solution was determined as described [28]. The extracellular solution was comprised of 150 mM NaCl, 10 mM HEPES, 3 mM KCl, 2 mM CaCl_2_, 2 mM MgCl_2_, and 5.5 mM glucose at pH 7.3 with NaOH (~ 320 mOsm).

### Yeast two-hybrid assay

The Best1 was ligated into pGBKT7 encoding for the GAL4 DNA binding domain (BD) and the 14–3-3γ was cloned into pGADT7 encoding for the activation domain (AD). To assess the protein–protein interaction between Best1 and 14–3-3γ, both BD/ mBest1 and AD/14–3-3γ were co-transformed into the yeast strain AH109. AH109 is unable to synthesize histidine. However, interaction between Best1 and 14–3-3γ enables the yeast to make the His3 enzyme, thereby permitting histidine biosynthesis and growth on His minimal medium.

### Construction of BEST1-deletion or S358A mutants

We used full length mouse Best1 cDNA which was cloned in our previous study [7] and cDNAs of 7 isoform of 14–3-3 were kindly provided from Dr. Dukryong Kim (Gyeongsang National University). For searching the binding site between BEST1 and 14–3-3γ, 4 kinds of C-terminal deletion mutant (C1, C2, C3 and ΔC1) and 1 point mutant (S358A) were generated by EZchange™ Site-directed mutagenesis kit (Enzymonics, Korea) and confirmed by sequencing.

### 14–3-3γ shRNA, and virus production

The 14–3-3γ nucleotides from 544 to 564 (5′-ggacaactacctgatcaagaa) were selected for target region of 14–3-3γ-shRNA. For shRNA expression, 14–3-3γ shRNA was synthesized as followings: 5′-t ggacaactacctgatcaagaattcaagagattcttgatcaggtagttgtccttttttc-3′ and 5′-tcgagaaaaaaggacaactacctgatcaagaatctcttgaattcttgatcaggtagttgtcca-3′. The annealed double stranded oligo was inserted into HpaI-XhoI restriction enzyme sites of pSicoR lentiviral vector (provided by Dr. T. Jacks; [29]) and verified by sequencing. Scrambled shRNA-containing pSicoR construct (control-shRNA) was used as control. To express shRNA with adenovirus in a Cre-dependent manner, pSicoR cassette containing U6 promoter, shRNA sequences and CMV-GFP flanked by loxP sites was transferred to pAd/CMV/V5-DEST plasmid (Invitrogen). Using these viral vectors, adenovirus was packaged at KIST Virus Facility (http://virus.kist.re.kr).

### Biotinylation assay

For surface biotinylation, 14–3-3γ-shRNA or control-shRNA infected cultured astrocytes were incubated at 4 °C and washed three times with PBS. Surface expressed proteins were then biotinylated in PBS containing sulfo-NHS-SS-biotin (Pierce) for 30 min. After biotinylation, cells were washed with quenching buffer (100 mM glycine in PBS) to remove excess biotin and then washed three times with PBS. The cells were then lysed and incubated with high capacity NeutrAvidin-agarose resin (Thermo science). After three washes with lysis buffer, bound proteins are eluted by SDS sample buffer and subjected to western blot analysis.

### Delivery of adenoviral vector containing 14–3-3γ-shRNA into mouse hippocampus

The adenovirus carrying 14–3-3γ-shRNA was injected into hippocampal CA1 region of CaMKIIα-Cre transgenic mouse brain. Because loxP-floxed shRNA in the adenoviral construct can be cleaved by Cre expression in the pSicoR system [29], delivering an adenoviral particle containing pSicoR-14-3-3γ*-*shRNA into CaMKIIα-Cre transgenic mouse brain allowed us to achieve CA1 pyramidal neuron-specific recovery of 14–3-3γ expression. After 2 weeks incubation, the mice were sacrificed for whole cell patch clamp recordings at 9 ~ 10 weeks of age. Only male mice were used in this study.

### Western blotting

Gene silencing of 14–3-3γ was tested by western blotting. To observe shRNA-mediated inhibition of 14–3-3γ expression, adenovirus carrying control-shRNA or 14–3-3γ-shRNA was infected to cultured astrocytes seeded on 35 mm dishes. After 72 h incubation, cells were lysed with RIPA buffer. 40 μg of proteins were separated by SDS–PAGE using 10% gels and blotted onto PVDF membranes. The blots were incubated overnight at 4 °C with anti-14-3-3γ antibody (1:500; Abcam). The blots were then washed and incubated with horseradish peroxidase-conjugated goat anti-mouse or anti-rabbit IgG, followed by washing and detection of immunoreactivity with enhanced chemiluminescence (Amersham Biosciences). The band intensity was acquired and analyzed by ImageQuant LAS 4000 (General Electric Company).

### Immunocytochemistry

The specificity of antibody (Abcam, rabbit polyclonal antibody 1:100) was tested by immunocytochemistry in cultured astrocytes in combination with 14–3-3γ-shRNA. Primary cultured astrocytes were transfected with 14–3-3γ-shRNA, grown on coverslips for additional 48 h. The cells were fixed in 4% paraformaldehyde for 30 min at room temperature, then permeabilized with PBS with 0.5% NP40 for 5 min. Non-specific binding was prevented with 1 h incubation in 2% donkey serums. Cells were incubated with the anti-14-3-3γ, and anti-GFAP (Millipore, chicken polyclonal antibody 1:500) primary antibodies for overnight at 4 °C. After washing, DyLight 488 or 649-conjugated secondary antibody (Jackson lab, 1:400) was added and incubated for 2 h at room temperature. The cells were washed and mounted, and then observed by confocal microscopy (Nikon A1). 3D reconstructions were generated from stacks of images with the confocal microscope software NIS-Elements.

### Co-immunoprecipitation

Flag-14-3-3γ and GFP-Best1 were co-expressed in COS7 cells and 24 h post-transfection, extracted with buffer (50 mM Tris-HCl, pH 7.4, 150 mM NaCl, 5 mM EDTA, 1 mM PMSF, and 1% NP-40) containing a protease-inhibitor cocktail. Whole cell lysates were incubated on ice for 30 min and then cleared at 20,000 g for 20 min at 4 °C and immunoprecipitated with Flag. After 2 h incubation at 4 °C, the beads were washed four times with ice cold phosphate-buffered saline (PBS). Bound proteins were eluted with SDS sample buffer, separated on 12% SDS–PAGE gels. The blots incubated overnight at 4 °C with anti-Flag antibody (1:1000; Santa Cruz Biotechnology) or anti-GFP antibody (1:1000; Abcam). Blots were then washed and incubated with horseradish peroxidase-conjugated goat anti-mouse or anti-rabbit IgG, followed by washing and detection of immunoreactivity with enhanced chemiluminescence (Amersham Biosciences) and captured by ImageQuant LAS 4000 (General Electric Company).

### Bimolecular fluorescence complementation (BiFC) experiment

Best1 WT, mutants (ΔC1, S358A) and 14–3-3γ were cloned into bimolecular fluorescence complement (pBiFC)-VN173 and pBIFC-VC155 vectors. HEK293T cells were transfected with each cloned BiFC vectors or co-transfected with cloned BiFC vectors in all possible pairewise combinations. These cells were fixed with 4% paraformaldehyde for 20 min at room temperature and washed twice with ice-cold phosphate-buffered saline (PBS). After fixation, the nuclei were stained with DAPI and mount with Dako fluorescence mounting medium. Venus fluorescence signals were observed by confocal microscopy (Nikon A1).

### Ca^2+^ imaging

For Ca^2+^ imaging, cultured astrocytes were incubated with 5 μM Fura-2 AM in 1 μM pluronic acid (Invitrogen) for 30 min at room temperature and subsequently transferred to a microscope stage for imaging using extracellular solution which was comprised of 150 mM NaCl, 10 mM HEPES, 3 mM KCl, 2 mM CaCl_2_, 2 mM MgCl_2_, and 5.5 glucose at pH 7.3 with NaOH (~320 mOsm). Intensity images of 510 nm wavelength were taken at 340 and 380 nm excitation wavelengths, and the two resulting images were taken for ratio calculations. Imaging Workbench software (INDEC BioSystems) was used for acquisition of intensity images and conversion to ratios.

### Electrophysiological recording from hippocampal slices

The brain from deeply anaesthetized mouse was rapidly removed and submerged in an ice-cold oxygenated artificial cerebrospinal fluid (ACSF) composed of 130 mM NaCl, 24 mM NaHCO_3_, 3.5 mM KCl, 1.25 mM NaH_2_PO_4_, 1.5 mM CaCl_2_, 1.5 mM MgCl_2_ and 10 mM glucose saturated with 95% O_2_ ~ 5% CO_2_, at pH 7.4. The hemisected brain was glued onto the stage of a vibrating microtome (Leica VT1000S) and sections of 300 μm thickness were cut and stored in an incubation chamber at room temperature for about 1 h before use. Slices were placed on the stage of an upright microscope underneath a nylon restraining grid, and superfused with oxygenated ACSF composed of 130 mM NaCl, 24 mM NaHCO_3_, 3.5 mM KCl, 1.25 mM NaH_2_PO_4_, 1.5 mM CaCl_2_, 5 μM MgCl_2_ and 10 mM glucose saturated with 95% O_2_ ~ 5% CO_2_, at pH 7.4.at room temperature (23 °C). The pipette solution for patching CA1 neuron contained 140 mM Cs-MeSO_4_, 10 mM HEPES, 7 mM NaCl, 4 mM Mg-ATP and 0.3 mM Na-GTP at pH 7.3 adjusted with CsOH (∼280 Osm). Visually guided whole-cell patch recordings were obtained from CA1 pyramidal neurons. 0.5 μM TTX and 20 μm bicuculline were added to the extracellular solution. Recordings were obtained using Axopatch 200A (Axon instruments) and filtered at 2 kHz. All described experimental procedures were performed in accordance with the institutional guidelines of Korea Institute of Science and Technology (KIST).

### Molecular modeling

The X-ray structures of a human 14–3-3γ protein (PDB code: 3UZD) and chicken Best1 (PDB code: 4RDQ) were retrieved from PDB bank (http://www.rcsb.org). The human (accessible code: O76090) and mouse Best1 (accessible code: O88870) amino acid sequences were obtained from the UniProt database for homology modeling. The sequence alignment of hBest1 or mBest1 with cBEST crystal structure was conducted by ClustalW implemented in DiscoveryStudio program (Accelrys). The 20 homology models on each species were generated using MODELLER in DiscoveryStudio program. The homology model of Best1 was selected with low PDF total energy. In chain A of both Best1 homology models, Ser358 residue was manually converted to phospho-Ser in DiscoveryStudio program and then the eight amino acids containing pSer358 were extracted from homology models (RRApS^358^FMGS for human; RRHpS^358^FMGS for mouse). 14–3-3γ protein and octa-peptide of Best1 were prepared with neutralization at pH 7.4 and energy minimization by Protein Prep Wizard in Maestro program (Schrodinger LLC). The grid box was automatically generated with peptide docking mode around HDAC4 peptide bound in 14–3-3γ protein. The Best1 peptides were flexibly docked into binding site of 14–3-3γ protein by Glide-SP Peptide module in Maestro program.

## Result

### 14–3-3γ specifically interacts with Best1

**Fig. 1:**
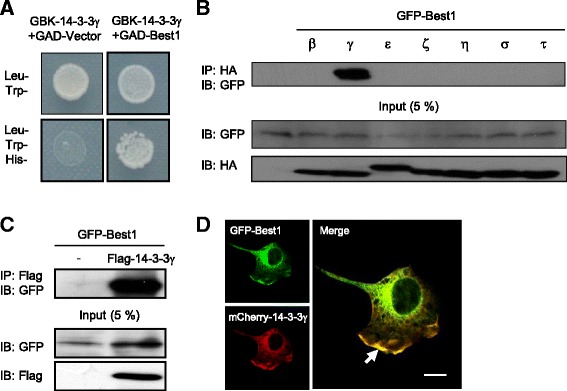
14–3-3γ interacts with Best1. **a** Identification of 14–3-3γ as a direct binding partner of Best1 in the yeast two-hybrid system. **b** Co-IP of GFP-Best1 with HA-tagged 14–3-3 isoforms in COS7 cells. Whole lysate of COS7 cells was immunoprecipitated with anti-HA antibody and then analyzed by Western blot with anti-GFP antibody. 5% of the total lysate was used as input for the immunoprecipitation. **c** Co-IP of Flag-14-3-3γ with GFP-Best1. Whole lysate of COS7 cells was immunoprecipitated with anti-Flag antibody and then analyzed by Western blot with anti-GFP antibody. 5% of the total lysate was used as input for the immunoprecipitation. **d** Representative confocal image of co-localization of Best1 and 14–3-3γ. GFP-tagged Best1 and mCherry-14-3-3γ were co-transfected in COS7 cell. Scale bar, 10 μm

To identify potential interacting partners for Best1, we performed conventional yeast-two hybrid (Y2H) and membrane Y2H screening using cytosolic N- and C-termini and full-length Best1 as bait. Among the positive clones, we identified 14–3-3γ as a binding partner protein of Best1. The potential interaction between these two proteins was further validated by using the full-length cDNAs of Best1 and mouse 14–3-3γ. We found that Best1 and 14–3-3γ formed a positive yeast colony in membrane Y2H under TLH- (the absence of Threonine, Leucine, and Histidine in yeast media) conditions, while the empty vector did not (Fig. 1a).

The 14–3-3 proteins are a family of conserved regulatory molecules in which seven mammalian isoforms (β, γ, η, ζ, ϵ, τ, and σ) are highly homologous and capable of binding to the same target. To examine the interaction between other 14–3-3 isoforms and mBest1 in a mammalian system, we constructed expression vectors for hemagglutinin (HA)-tagged 14–3-3β, γ, η, ζ, ϵ, τ, and σ (HA–14-3-3β, γ, η, ζ, ϵ, τ, and σ) and co-expressed with GFP–mBest1 in COS7 cells. We then performed co-immunoprecipitation (Co-IP) on cell lysates with an anti-HA antibody and then blotted with an anti-GFP antibody. The results showed that GFP–Best1 was associated only with HA–14-3-3γ (Fig. 1b). The association of GFP-Best1 with 14–3-3γ was reconfirmed when we used Flag-tagged 14–3-3γ (Flag–14-3-3γ) and GFP–Best1 in COS7 cells (Fig. 1c). To test whether such interactions between Best1 and 14–3-3γ can be observed in COS7 cells, we also examined the expression pattern of GFP-tagged Best1 and mCherry-tagged 14–3-3γ in these cells. We observed a strong co-localization of Best1 and 14–3-3γ proteins (in yellow color) particularly in the plasma membrane rich regions of transfected COS7 cells as indicated by an arrow (Fig. 1d). These results indicate that among seven 14–3-3 isoforms, 14–3-3γ isoform specifically interacts with Best1.

### C-terminus of Best1 is critical for binding with 14–3-3γ

**Fig. 2:**
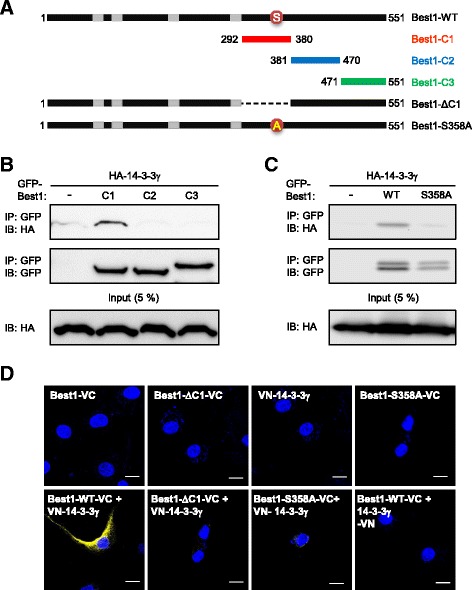
C-terminus of Best1 is important for binding with 14–3-3γ. **a** Schematic diagram of full-length (Best1-WT), the first C-terminal region (292–380 amino acids, Best1-C1), the second C-terminal region (381–470 amino acids, Best1-C2), the third C-terminal region (471–551 amino acids, Best1-C3), C1 deleted mutant of Best1 (Best1-ΔC1) and S358A mutant of Best1 (Best1-S358A). **b** Co-IP of HA-14-3-3γ with GFP-Best1-C1 (C1), GFP-Best1-C2 (C2) and GFP-Best1-C3 (C3). Whole lysate of COS7 cells was immunoprecipitated with anti-HA antibody and then analyzed by Western blot with anti-GFP antibody. 5% of the total lysate was used as input for the immunoprecipitation. **c** Co-IP of HA-14-3-3γ with GFP-Best1-WT (WT) and GFP-tagged S358A mutant of Best1 (S358A). Whole lysate of COS7 cells was immunoprecipitated with anti-HA antibody and then analyzed by Western blot with anti-GFP antibody. 5% of the total lysate was used as input for the immunoprecipitation. **d** BiFC experiment with VN-14-3-3γ and Best1-VC, where N- and C-terminal halves of Venus fluorescent protein were fused to 14–3-3γ and Best1, respectively. BiFC fluorescent signals were detected by the yellow color. The fluorescent signal was detected at the plasma membrane of the cells by the yellow color. Cells only expressing both Best1-WT-VC and VN-14-3-3γ showed intense fluorescent signal (lower left panel). Scale bar, 10 μm

To investigate which part of Best1 contributes to binding with 14–3-3γ protein, we predicted the putative phosphorylation sites in Best1 amino acid sequence for binding with 14–3-3γ through the web server that allows users to predict 14–3-3-binding sites in a protein of interest [http://www.compbio.dundee.ac.uk/1433pred/]. Because it was proposed that Bestrophin’s C-termini are involved in protein-protein interaction [9], 14–3-3γ binding motif probably resides on the C-terminus of Best1, which is known to be a cytosolic domain according to the recently reported crystal structure of Best1 [30, 31]. To test this possibility and to determine the minimum 14–3-3γ-binding domain, we generated a series of GFP-tagged 14-3-3γ construct by subdividing Best1-C into three parts (Fig. 2a), which are Best1-C1 (292–380 amino acid residues), Best1-C2 (381–470 amino acid residues) and Best1-C3 (471–551 amino acid residues). We then performed Co-IP on cell lysates with an anti-GFP antibody and then blotted with an anti-HA antibody (Fig. 2b). We found that 14–3-3γ interacted with Best1-C1, but not with Best1-C2 or Best1-C3. It is generally accepted that a number of binding partners for 14–3-3 share a common binding determinant that mediates their contact with 14–3-3. Several reports demonstrated that phosphorylation of target protein is the primary mechanism that controls 14–3-3 binding [17]. A few conserved phosphorylation sites were suggested as consensus 14–3-3 recognition motif [22, 32, 33]. There is a putative 14–3-3γ binding motif RRHpSF which contains serine phosphorylation site (S358) within the Best1-C1 of Best1. To test whether this S358 of Best1 is the critical residue necessary for 14–3-3γ binding, we replaced S358 of GFP-tagged Best1 to alanine (S358A). Co-IP experiment revealed a drastic reduction in binding of S358A mutant of Best1 with 14–3-3γ (Fig. 2c), suggesting that phosphorylation at S358 at Best1 is critical for the interaction with 14–3-3γ.

We then examined the interaction between Best1 and 14–3-3γ at the single-cell level by bimolecular fluorescence complementation (BiFC) assay, which allows visualization of two independent proteins in close spatial proximity [34]. We constructed Best1 and 14–3-3γ, whose N- and C- termini were fused with one complementary halves of split Venus fluorescent protein, either N-terminal half (VN) or C-terminal half (VC), and then both were transfected into HEK293T cells (Fig. 2d). Strong yellow fluorescence was detected when the split Venus halves were on complementary positions (Best1-VC and VN-14-3-3γ, Fig. 2d, lower left). In contrast, very weak or virtually no BiFC signal was detected from the cells transfected with VN-14-3-3γ and Best1-ΔC1-VC which is a C1 deletion mutant of Best1 (Fig. 2a) fused to VC (Fig. 2d, lower second). In addition, we also tested Best1-S358A-VC (Fig. 2d, lower third) and found no BiFC signal with VN-14-3-3γ. As a negative control, when Best1 or 14–3-3γ was expressed with only one half of split Venus, no BiFC fluorescence was detected (Fig. 2d, upper panels). Taken together, the C-terminal domain (292–380 amino acid residues) of Best1 is important for binding with 14–3-3γ and potential phosphorylation site S358 within this region is critical for the interaction with 14–3-3γ.

### Phospho-Serine358 residue of Best1 interacts with 14–3-3γ

**Fig. 3:**
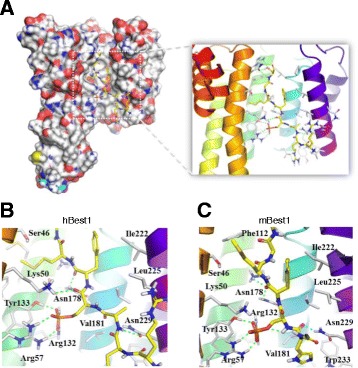
Predicted interaction mode of the putative 14–3-3γ binding site of Best1. **a** 14–3-3γ in complex with a phospho-peptide of Best1. Closed-up view of binding motif of hBest1 (**b**) and mBest1 (**c**). 14–3-3γ (PDB: 3UZD) recognize the putative binding motif of hBest1 and mBest1. Molecular surface of 14–3-3γ represented with charge of residues. The residues of 14–3-3γ involved in the binding are shown as white sticks. The segment of hBest1 and mBest1 display with yellow-colored stick. The secondary structure of 14–3-3γ are depicted with cartoon

We carried out the peptide docking based on the molecular modeling to predict the binding mode of the putative 14–3-3γ binding motif of Best1 with 14–3-3γ (PDB: 3UZD) [35]. The putative 14–3-3γ binding motif RRApS^358^FMG of human Best1 (hBest1) and RRHpS^358^FMG of mBest1, identified by mutation study in Fig. 2, were well-fitted to central groove of 14–3-3γ (Fig. 3a). The interaction and orientation of hBest1 and mBest1 were stabilized by charge interactions of Arg57-Arg132-Tyr133 triad of 14–3-3γ with pSer358 of Best1, and backbone hydrogen bonding of Lys50, Asn178 and Asn229 residues (Fig. 3b, c). Hydrophobic side chains of hBest1 and mBest1 interacts with Ile222, Leu225 and Trp233 of hydrophobic groove of the 14–3-3γ. On the other side of its groove, Met360 occupied the charged region of 14–3-3γ, composed of Ser46, Lys50, and Asn178. This docking simulation provided structural binding details of putative binding motif of Best1 that the segment containing pSer358 of Best1 is an essential for 14–3-3γ recognition and binding.

### 14–3-3γ-shRNA reduced the Best1 current and membrane expression in astrocyte

**Fig. 4:**
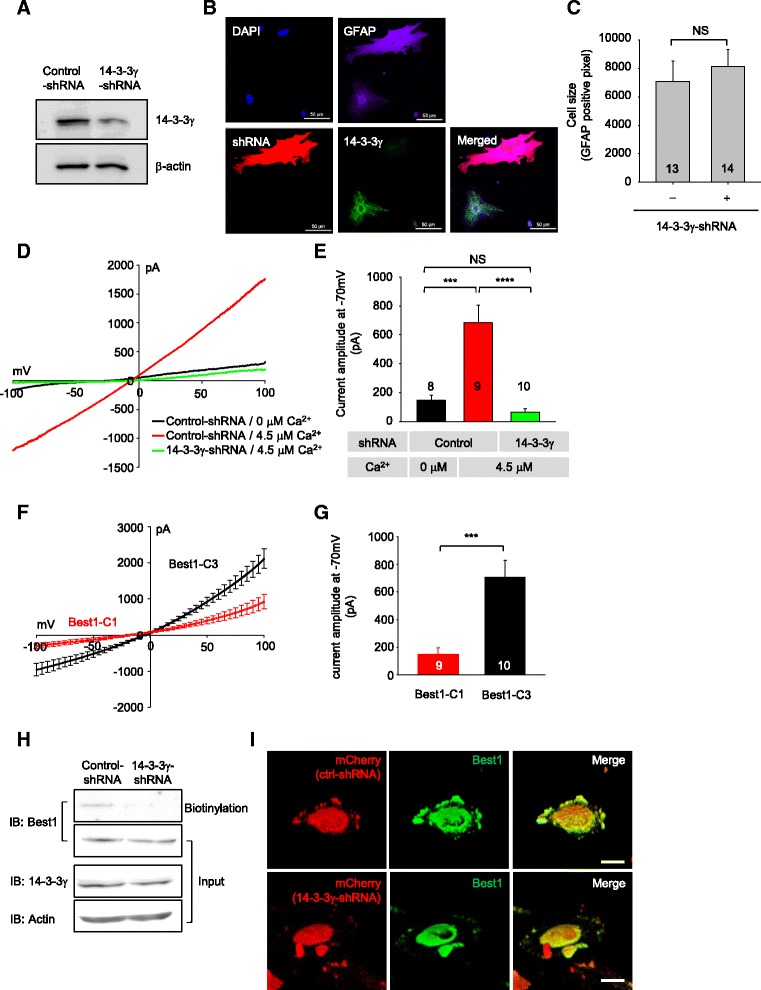
14–3-3γ -shRNA reduced the expression of 14–3-3γ and membrane expression of Best1 in astrocyte. **a** 14–3-3γ shRNA reduced the level of 14–3-3γ expression mRNA in cultured astrocytes as determined by Western blot. Cultured astrocytes were transfected with pSicoR-control-shRNA or pSicoR-14-3-3γ-shRNA and β-actin was used as an internal control. **b** Confocal immunofluorescence images of cultured astrocytes transfected with pSicoR-14-3-3γ-shRNA-mCherry. Scale bar, 50 μm. **c** Quantification of the two-dimensional cell area by counting the number of GFAP positive pixel for the experiment described in (**a**). Numbers of determinations are indicated on the bar graph. NS *p =* 0.5726 (unpaired two-tailed t test). **d** Representative I–V responses of cultured astrocyte expressing pSicoR-control-shRNA or pSicoR-14-3-3γ-shRNA under whole-cell patch-clamp configuration using 4.5 μM Ca^2+^-containing or 0 Ca^2+^-containing patch pipette solution. **e** Bar graph showing summary of current amplitudes (mean ± SEM). The current responses were recorded in response to a voltage ramp command (from −100 to +100 mV, 1 s duration, 0.2 Hz; V_h_ of −70 mV). Numbers of determinations are indicated on the bar graph. Asterisk indicates a significant difference determined by one-way ANOVA test and Tukey’s multiple comparison test (****p < 0.0001, ***p = 0.0001, NS *p* = 0.7227). **f** Averaged I–V responses of cultured astrocytes expressing Best1-C1 or Best1-C3 under whole-cell patch-clamp configuration using 4.5 μM Ca^2+^-containing patch pipette solution. **g** Bar graph showing summary of current amplitudes (mean ± SEM). The current responses were recorded in response to a voltage ramp command (from −100 to +100 mV, 1 s duration, 0.2 Hz; V_h_ of −70 mV). Numbers of determinations are indicated on the bar graph. Asterisk indicates a significant difference determined by unpaired two-tailed t-test (***p < 0.001). **h** Cell-surface biotinylation assay in cultured astrocytes. When astrocytes transiently transfected with 14–3-3γ-shRNA, surface expression of Best1 is dramatically decreased. **i** Cultured astrocytes transfected with pSicoR-control-shRNA-mCherry (upper panel) or pSicoR-14-3-3γ-shRNA-mCherry (bottom panel) were imaged by confocal microscopy using antibodies against Best1 and 3D reconstructions were generated from stacks of images. Scale bars, 20 μm

Astrocytes have been shown to express five isoforms of 14–3-3 by RT-PCR: β, γ, η, ξ, and ε [36]. To determine the function of 14–3-3γ in regards to Best1 in astrocyte, a 14–3-3γ-specific short hairpin RNA (shRNA, 14–3-3γ-shRNA) was designed and tested to selectively knockdown the expression of 14–3-3γ transcript. Western blot analysis showed that the expression of 14–3-3γ-shRNA resulted in about 57% knockdown of endogenous 14–3-3γ protein in cultured astrocytes (Fig. 4a). Considering that the efficiency of cell transfection with pSicoR-control-shRNA or pSicoR-14-3-3γ-shRNA was around 60%, about 90% of knockdown of 14–3-3γ expression was achieved by 14–3-3γ-shRNA. We also confirmed that cultured astrocyte transfected with pSicoR-14-3-3γ-shRNA-mCherry showed an effective knockdown of endogenous 14–3-3γ protein level (Fig. 4b) without any change of cellular morphology or size (Fig. 4c) by immunocytochemistry.

To test whether gene-silencing of 14–3-3γ affects astrocytic Best1 current, we subsequently recorded astrocytic Best1 current in control-shRNA- or 14–3-3γ-shRNA-expressing cultured astrocytes using whole-cell patch clamp recording, as previously described [37]. We directly induce an increase in conductance in astrocytes by a membrane rupture for whole-cell mode with internal solutions containing high free [Ca^2+^]_i_ (~ 4.5 μM). We found that Ca^2+^-activated Best1 current in cultured astrocyte was significantly suppressed by 14–3-3γ-shRNA expression (Fig. 4d, e, current amplitude at *V*_h_ = −70 mV; control-shRNA-expressing astrocytes, 684 ± 121 pA, *n* = 9; 14–3-3γ-shRNA-expressing astrocytes, 67 ± 23 pA, *n* = 10; ****p < 0.0001 vs control-shRNA group; one-way ANOVA and Tukey’s multiple comparison test). The control group astrocytes expressing control-shRNA recorded with no free [Ca^2+^]_i_ –containing internal solutions did not show any significant current as expected (Fig. 4d, e, current amplitude at *V*_h_ = -70 mV; 148 ± 34 pA, *n* = 8).

To test the functional consequence of the interaction between the C-terminus of Best1 and 14–3-3γ, we over-expressed Best1-C1 and recorded Ca^2+^-activated Best1 current in cultured astrocytes. We found that Best1 current amplitude was significantly reduced by the over-expression of Best1-C1 in astrocytes compare to that of Best1-C3 which is not capable of interacting with 14–3-3γ (Fig. 4f, g, current amplitude at *V*_h_ = −70 mV; Best1-C1-expressing astrocytes, 148.7 ± 46.8 pA, *n* = 9; Best1-C3-expressing astrocytes, 705.6 ± 122.6 pA, *n* = 10; ***p = 0.0008 vs Best1-C1 group; unpaired two-tailed t-test). This set of results is explained by a competition between endogenous Best1 and over-expressed Best1-C1 to interact with 14–3-3γ.

14–3-3γ-shRNA could reduce surface expression of Best1 in astrocyte. To test the function of 14–3-3γ in surface expression of Best1 in astrocyte, we performed a surface biotinylation assay in cultured astrocyte. Treatment with 14–3-3γ-shRNA caused a dramatic reduction in surface expression of the endogenous Best1 channel proteins, measured by surface biotinylation, in comparison to control-shRNA treatment, without affecting the total Best1 protein levels in astrocytes (Fig. 4h). We also examined the surface expression of Best1 by immunocytochemistry to determine the effect of 14–3-3γ shRNA on the subcellular localization of Best1 (Fig. 4i). Best1 channels were highly localized at the plasma membrane of cultured astrocytes in the presence of control-shRNA, while they were less localized at the plasma membrane in the presence of 14–3-3γ shRNA. These results demonstrate that 14–3-3γ promotes the surface expression of Best1 by interaction with C-terminus of Best1 in astrocytes.

### 14–3-3γ shRNA reduced Best1 mediated glutamate release from hippocampal astrocyte

**Fig. 5:**
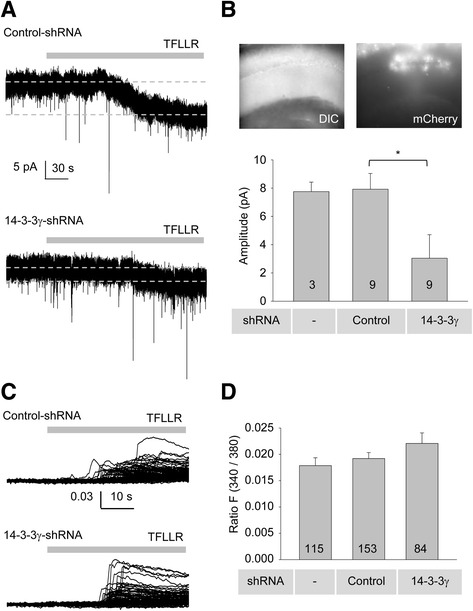
14–3-3γ-shRNA reduced Best1 mediated glutamate release from hippocampal astrocyte. **a** Representative measurement of glutamate current in a somatic region of CA1 pyramidal neuron of CaMKIIα-Cre mouse injected with adenovirus expressing control-shRNA or 14–3-3γ-shRNA under voltage clamp held at −60 mV during treatment with TFLLR (30 μM, grey bar). **b** Upper figures indicate fluorescent protein mCherry images of hippocampal CA1 region injected with adenoviruses transducing either control-shRNA or 14–3-3γ-shRNA. Bar graphs represent the averaged amplitudes (differences between baseline and TFLLR induced current amplitude depicted as dashed lines in (**a**)). Numbers of tested slices from at least two independent mice are indicated in the bar graph. Asterisk indicates a significant difference determined by one-way ANOVA test (*p < 0.05). **c** Traces from Ca^2+^ imaging recordings performed in naive astrocytes, pSicoR-control-shRNA expressing astrocytes, or pSicoR-14-3-3γ-shRNA expressing astrocytes. Each trace represents a Ca^2+^ response in one cell during treatment with TFLLR (30 μM, grey bar). **d** Summary bar graph for calcium imaging. Numbers of recorded cells are indicated on the bar graph

We have previously demonstrated that astrocytes release glutamate upon GPCR activation via Best1 [13]. We also demonstrated that the target of Best1-mediated astrocytic glutamate is the synaptically localized, NR2A containing NMDA receptors (NMDAR) in hippocampal CA1 pyramidal neurons [11, 12]. To test whether 14–3-3γ contributes to astrocytic glutamate release via Best1, we directly measured the effects of astrocyte-specific knocking down of 14–3-3γ in vivo by introducing adenovirus carrying 14–3-3γ*-*shRNA into hippocampal CA1 region of CaMKIIα-Cre mice (see Methods). To induce astrocytic glutamate release, we used 30 μM TFLLR, PAR1 agonist applications to activate PAR1, which is known to be expressed mostly in CA1 hippocampal astrocytes. We applied to whole brain slices by perfusion and recorded TFLLR-induced whole cell current at −60 mV from CA1 pyramidal neuron in hippocampal slices prepared from adenovirus-injected mouse brain in the presence of tetrodotoxin (TTX) and Bicuculline. Compared to the control-shRNA, 14–3-3γ-shRNA markedly reduced TFLLR-induced astrocytic glutamate release via Best1 (Fig. 5a, b, current amplitude at *V*_h_ of −60 mV; control-shRNA-expressing astrocytes, 7.7 ± 1.0 pA, *n* = 10; 14–3-3γ-shRNA-expressing astrocytes, 3.0 ± 1.7 pA, *n* = 9; *p = 0.0413 vs control-shRNA group; one-way ANOVA test). As we have shown in previous studies [4, 13], astrocytic PAR-1 mediated glutamate release induced by TFLLR was APV sensitive (data not shown). 14–3-3γ-shRNA-expressing cultured hippocampal astrocytes did not show any difference in TFLLR-induced Ca^2+^ increase compared to control-shRNA-expressing astrocytes (Fig. 5c, d). These results demonstrate that 14–3-3γ can regulate the surface expression of Best1 in vivo.

## Discussion

We have identified strong interaction between Best1 and 14–3-3γ and the interaction is γ isoform specific among seven 14–3-3 isoforms. Using truncated Best1 and Best1 mutant, we have shown that 292–380 amino acids of the C-terminus contribute to the interaction between the channel and the 14–3-3γ and Ser358 residue of Best1 is critical for this interaction. The binding motif was further narrowed down by molecular modeling to be RRApS^358^FMG of hBest1 and RRHpS^358^FMG of mBest1. The most striking finding was that gene-silencing of 14–3-3γ expression in astrocytes disrupted (i) the expression of Best1 functional current in cultured astrocytes, (ii) the surface expression of Best1 in cultured astrocytes, and (iii) PAR-1 induced NMDA current in hippocampal CA1 pyramidal neuron. It raises the possibility that 14–3-3 may be involved in post-translational regulation of channel expression, for example, by participating in channel assembly in the endoplasmic reticulum or by modulating trafficking, retention or retrieval of Best1 channel proteins.

It is possible that through its interaction with Best1, 14–3-3γ modulates gliotransmission not only under physiological condition but also under pathological condition by regulating the surface expression of Best1 in astrocytes. There are numerous reports showing that 14–3-3 proteins have been involved in the pathophysiology of various neurological disorders [19]. 14–3-3γ level is significantly decreased in the cortex of embryos with Down’s syndrome and is elevated in several brain regions of patients with AD and in Lewy bodies in Parkinson’s disease brains [38]. The elevated level of 14–3-3γ expression in the brain of AD patients could explain the change of Best1 localization from microdomains to the soma and processes in reactive astrocytes [14]. Therefore, the changed expression level of 14–3-3γ in neurological disorders might lead to a possible impairment of synaptic plasticity and signaling pathways which are likely to correlate with transcriptional control or signal transduction, as well as modulation of gliotransmission. Determining the role of 14–3-3 proteins in brain function may lead to advances in understanding how these proteins affect neurological disorders.

Bestrophins including hBest1 and mBest1 have predicted high-stringency phosphorylation sites (scansite.mit.edu) for protein kinase A (PKA), protein kinase C (PKC), and various other kinases. hBest1 interacts physically and functionally with protein phosphatase 2A [39]. Taken together, phosphorylation may play an important role in regulation of surface expression of Bestrophins. Interestingly, putative 14–3-3γ binding motif of Best1 which was tested in this study shares a putative protein kinase C (PKC) phosphorylation site (pSFMGS). It is possible that 14–3-3γ provides a link between Best1 and PKC. Future studies are needed to better understand the potential kinases and phosphatases that are involved in interaction of 14–3-3γ for regulating physiological function of Best1.

## Conclusion

In summary, we have provided the first evidence for controlling mechanism for the surface expression of Best1 channels in astrocytes. The regulation of the surface expression of Best1 via 14–3-3γ binding may represent an important determinant of the neuromodulation both in physiological and pathological conditions.
